# Citric Acid Improves Egg White Protein Foaming Characteristics and Meringue 3D Printing Performance

**DOI:** 10.3390/foods14020198

**Published:** 2025-01-10

**Authors:** Huajiang Zhang, Shihui Hua, Mengzhuo Liu, Rui Chuang, Xin Gao, Hanyu Li, Ning Xia, Chaogeng Xiao

**Affiliations:** 1 College of Food Science, Northeast Agricultural University, Harbin 150030, China; hjthzhang@163.com (H.Z.); 18841561653@163.com (S.H.); 19997797983@163.com (M.L.); ruichuang2004@163.com (R.C.); lihanyu1004@126.com (H.L.); xianing1981@126.com (N.X.); 2 Food Science Institute, Zhejiang Academy of Agricultural Sciences, Hangzhou 310021, China; xiaochaogeng@163.com

**Keywords:** egg whites, citric acid, foaming property, meringue, rheological property, 3D printing

## Abstract

Meringue has limited the use of meringue for personalization because of its thermally unstable system. Citric acid (CA) enhancement of egg white protein (EWP) foaming properties is proposed for the preparation of 3D-printed meringues. The results showed that CA increased the viscosity, exposure of hydrophobic groups (79.8% increase), and free sulfhydryl content (from 5 µmol/g to 34.8 µmol/g) of the EWP, thereby increasing the foaminess (from 50% to 178.2%). CA treatment increased the rates of adsorption, stretching, and orientation of EWP at the air–water interface to form multiple layers, resulting in a delay in foam thinning. The secondary structure of CA-treated EWP remained intact, and the exposure of amino acid residues in the tertiary structure increased with the expansion of the hydrophobic region. CA-treated EWP-prepared protein creams had a suitable viscosity (from 233.4 Pa·s to 1007 Pa·s at 0.1 s^−1^), shear thinning, structural restorability, and elasticity, which ensured good fidelity of their printed samples. Experiments involving 3D printing of CA-treated EWP showed that CA could significantly enhance the 3D printing fidelity of EWP. Our study could provide new ideas for the development of customizable 3D-printed foam food products.

## 1. Introduction

Foam properties are influenced mainly by foaming capacity and foam stability. Foaming capacity is the key to improving the supporting properties and productivity of foam products, with the main influence coming from the protein adsorption layer [1]. When proteins can rapidly diffuse into the air–water interface and transform into an adsorbed state, their foaming capacity is greatly enhanced. Foam stability can maintain product appearance and prevent foam collapse [2]. Foam drainage, disproportionation, and coalescence may all contribute to foam instability. Drainage is the flow of liquid out of foam under the influence of surface tension and gravity, resulting in a change in the foam from a spherical structure to a polygonal structure and a consequent thinning of the liquid film. Disproportionation occurs when there is a gap in the internal pressure between the bubbles, leading to the extravasation of gasses from liquid film, and at this stage, an increase in size of the large bubbles and a continuous shrinkage of small bubbles until they disappear can be observed together. Coalescence is caused by the rupture of the liquid film, which leads to the merging of two neighboring bubbles into one larger bubble, thereby exacerbating the decrease in the number of bubbles [3].

Egg white proteins (EWPs) have both hydrophilic and hydrophobic properties, exhibit excellent foaming properties (foaming capacity and foam stability), and are widely used in the food industry for bakery products such as marzipan, sponge cake, and mousse cake [4]. As egg white powder is more suitable for long-term storage and long-distance transport than fresh egg whites are, it is widely used in practical production [5]. However, the instantaneous high temperatures during the drying process, when fresh egg whites are transformed into EWP, lead to a decrease in foaming properties of proteins, which limits the application of EWP in the food industry [6]. Therefore, improving the foaming properties of egg white proteins is important for improving quality of protein-containing foods. At present, various physical or chemical methods have been developed to enhance the foaming properties of EWP, but most of the methods are expensive and cumbersome, so an economical, simple, and efficient method should be proposed [7]. Acidic induction is a simple and efficient way to modify foaming properties of proteins. When proteins are exposed to extremely acidic or alkaline conditions, the extreme environment preserves the secondary structure of protein and destroys its tertiary structure. Proteins are converted into a more structurally flexible and more amphipathic molten globule (MG) to improve their foaming properties [8]. In addition, acidic induction can affect the interfacial properties of proteins by modulating their structure. Proteins in their natural state have a compact structure, small molecular flexibility, and low surface hydrophobicity, and cannot be quickly adsorbed to the air–water interface, so their foaming characteristics are poor. In a polar environment, the protein structure unfolds and folds, modifying the protein structure into a disordered state, improving hydrophobicity and flexibility, and increasing the interfacial properties of the protein [9]. However, the commonly used method of creating extreme environments with hydrochloric acid has safety concerns [10,11]. Citric acid (CA) is a multifunctional and important organic acid that is widely used as an acidifier, pH regulator, flavor enhancer, and antioxidant booster [12,13]. The use of citric acid as an acidifier enhances the safety attributes of the food and improves the flavor of the finished meringues. Therefore, we proposed a hypothesis to replace traditional acidifiers such as hydrochloric acid with citric acid to induce egg white protein, expand its structure, improve air–water interface properties, and enhance the foam performance of EWP, thereby increasing the application potential of EWP-stabilized foam and provide new possibilities for the production of edible foam.

Meringue is an aerated food product prepared by mixing egg whites and granulated sugar as the main ingredients at high speed, usually presenting a soft/hard peak-like batter. It is a very popular confectionery baking product and the basis for other complex baking products, such as mousse cakes, macaroons, and angel food cakes [14]. Meringue has more than 80% air-phase components, so its properties are mainly controlled by the foam structure properties and stability, and it forms its unique peak paste through the behavioral changes in the building and destruction of the foam structure [15]. Currently, there are few studies related to meringues, and the homogenization of existing meringues is serious; thus, there is an urgent need to develop new types of meringues with high foaming properties and personalized meringues. Three-dimensional printing (3D printing), also known as additive manufacturing and rapid prototyping, is an emerging digital manufacturing technology that constructs 3D objects by accumulating materials layer by layer [16]. Unlike traditional reduction material manufacturing (cutting), 3D printing eliminates the need to remove material and instead builds 3D objects from digital models, providing easy access to personalized food design. A variety of food substrates, such as chocolate [17], biscuit dough [18], meat gels [19], and vegetables [20], have been used to print 3D food products. These materials modulate the rheological properties of 3D printing inks, thereby influencing their flow behavior and printability. They can control the viscosity and storage modulus of the ink through solid–solid intermolecular interactions. However, for meringue 3D printing inks, solid–air interactions, i.e., air–water interfacial properties, are mainly involved. This places greater demands on the rheological behavior of meringue inks in terms of the viscosity and modulus, especially in terms of foaming and stability of the foam inks. The deformation resistance and uniform distribution of the foam are conducive to the structure of the foam is not easy to be destroyed in the 3D printing process, and has good recovery performance, which enhances the reduction degree of the 3D printing model.

In this study, an ink made of citric acid-modified EWP was developed for the production of 3D-printed meringues. The structural and air–water interfacial properties of EWP-CA were influenced by controlling the amount of citric acid added. The aim was to address the challenge of foam edibility and to offer the possibility of personalized meringue preparation. This study addresses the complexity of 3D-printable foams and provides a new perspective on edible use of protein foaming properties.

## 2. Materials and Methods

### 2.1. Materials

Egg white protein powder was purchased from Heilongjiang Zhongnong Xinghe Biotechnology Co., Ltd. (Harbin, China), citric acid was purchased from the Tianda Chemical Reagent Factory (Tianjin, China), and 8-aniline-1-naphthalenesulfonic acid (ANS) was purchased from Aladdin Biochemistry Science and Technology Ltd. (Shanghai, China). Other reagents were level analysis reagents.

### 2.2. Sample Preparation

EWP was dissolved in deionized water, magnetically stirred for 2 h, and stored at 4 °C overnight to ensure complete protein hydration. After the protein concentration was adjusted to 10 mg/mL, citric acid was added in different proportions so that the concentration of citric acid in the solution was 2 mg/mL, 5 mg/mL, 10 mg/mL, or 15 mg/mL, and the EWP solution without citric acid was used as a control. All samples were heated in a water bath at 50 °C for 5 h (the pH values of solutions containing citric acid after a water bath were approximately 3.4, 3.2, 2.9, and 2.5, respectively). The pH was uniformly adjusted back to 3.5 with NaOH (1 mol/L) to obtain EWP-CA complexes (EWP-CA-0, EWP-CA-0.2, EWP-CA-0.5, EWP-CA-1.0, and EWP-CA-1.5). The samples were then freeze-dried and stored. The freeze-dried powder was dissolved in deionized water and configured into a 10 mg/mL solution for subsequent characterization experiments.

### 2.3. Foam Characteristics

#### 2.3.1. Foaming Capacity and Foam Stability

The foaming properties of the samples were investigated according to previous methods [21]. In total, 10 mL of EWP dispersion (10 mg/mL) was stirred for 2 min at 25 °C using a homogenizer (Olympus Co., Ltd., Tokyo, Japan) at 10,000 r/min. The whipped samples were left to stand for 1 min, and the volume of foam in the measuring cylinder was read. The foaming capacity (FC) and foam stability (FS) were calculated via Equations (1) and (2).

(1) $$FC=\frac{V_{0}}{V_{L}}\times 100\%$$

(2) $$FS=\frac{V_{30}}{V_{0}}\times 100\%$$

where V_L_ is the volume of the solution before homogenization; V_0_ and V_30_ are the volumes of foam after 1 min and 30 min of homogenization, respectively.

#### 2.3.2. Morphology of Foam

An appropriate amount of foam was taken at the center of the slide, and the foam morphology was observed under a 4 × objective lens via a research-grade ortho-microimaging system (Olympus Co., Ltd., Tokyo, Japan). All foam samples were stored at 25 °C, and the changes in the shape and size of the foam were recorded at 0 min, 2 min, 10 min, 30 min, and 60 min, respectively.

### 2.4. Rheological Viscosity

The viscosity of the samples was studied via a MARS 40 rheometer (Thermo Scientific, Waltham, MA, USA). We followed the previous measurement method and made slight modifications, removed 3 mL of the sample solution, and placed it on the rheometer plate [22]. The probe diameter was 60 mm, the measuring gap height was 1 mm, and the shear rate range was 0.1–100 s^−1^. Subsequently, the power-law equation was for fitting.

(3) $$\eta =k_{1}\cdot \gamma^{n_{1}-1}$$

where k_1_ represents the coefficient of consistency and n_1_ represents the flow property index.

### 2.5. Surface Hydrophobicity

The protein surface hydrophobicity of the complex egg white solutions was determined according to previous methods with slight modifications [23]. At room temperature, EWP-CA dispersions were diluted with deionized water (pH = 3.5) to obtain seven successive concentrations of protein levels in the range of 0.01–0.5 mg/mL. Afterwards, 80 µL of the ANS solution (8 mM) was added to each sample (8 mL), vortexed for 10 s, and incubated in the dark for 10 min. Fluorescence intensity (FI) was measured via an F-7100 fluorescence spectrophotometer (Hitachi, Tokyo, Japan) with the following experimental parameters: an excitation wavelength of 390 nm, emission wavelength range of 400–600 nm, slit width of 5 nm, and voltage of 400 V. H_0_ was expressed as the slope of the linear regression of the fluorescence intensity against protein concentration.

### 2.6. Free Sulfhydryl Groups

The free sulfhydryl groups of the samples were measured with some modifications based on a previous method [24]. The samples were diluted to a concentration of 2 mg/mL with a Tris-Gly (0.04 M EDTA, 0.09 M Gly, 0.086 M Tris, pH 8.0) buffer. In total, 3 mL of the sample was mixed with 0.03 mL of Ellman’s reagent (4 mg/mL DNTB Tris-Gly buffer), mixed thoroughly, and then reacted in the dark for 15 min at 25° C. The absorbance at 412 nm was obtained with a UV-6100 UV-Spectrophotometer (Shimadzu Instrument Co., Ltd., Suzhou, China). The formula for calculating the amount of free sulfhydryl groups was as follows:

(4) $$-SH(µmol/g)=\frac{73.53\times A\times D}{C}$$

where C is the original protein concentration, A is absorbance, and D is the dilution factor, where 73.53 = 10^6^/1.36 × 10^4^, where 1.36 × 10^4^ is the molar extinction coefficient.

### 2.7. Measurement of Interfacial Tension

We referenced previous methods [25]. The dynamic surface tension of the EWP solutions was determined via a video–optical goniometer (Theta, Biolin Scientific, Gothenburg, Sweden). The EWP solution was used as an aqueous phase at 25 °C. The interfacial tension between the aqueous phase (EWP solution) and air phase was measured, and 5 μL of the sample solution was injected into the air via a 25 μL injector while the digital camera captured the droplet shape. The adsorption process was recorded for 600 s, and the data were fitted via the Laplace–Young equation.

When the molecular diffusion phase is complete, the molecules will enter the permeation rearrangement phase. The permeation rate constant K_P_ and rearrangement rate constant K_f_ can be obtained by using Equation (5) to draw a curve, and then by linear fitting:

(5) $$ln[(\pi_{600}-\pi_{t})/(\pi_{600}-\pi_{0})]=-k_{i}t$$

where π_600_, π_0_, and π_t_ are the interfacial pressures at 600 s, t s, and 0 s, and k_i_ is the first-order rate constant.

### 2.8. Fourier Transform Infrared (FTIR) Spectroscopy

The FT-IR measurement method followed a previous method, with some modifications [26]. At room temperature, the complexes were freeze-dried and ground into powder, which was homogeneously mixed with KBr powder in the ratio of 1:100 (*w*:*w*) and pressed into tablets. Infrared spectra in the range of 400–4000 cm^−1^ were recorded via a Fourier transform infrared spectrometer. The detection parameters were set to a resolution of 4 cm^−1^ and 32 results were recorded. An amide I band (1600–1700 cm^−1^) was used to analyze the secondary structure content of the proteins via Peak Fit software (version V4.12).

### 2.9. Fluorescence Spectral Analysis

The fluorescence emission spectra of samples were recorded via an F-7100 fluorescence spectrophotometer (Hitachi, Japan). The intrinsic fluorescence spectrum of the sample solution (0.4 mg/mL) at 25 °C was excited at a wavelength of 280 nm and scanned in the range of 300–500 nm [27]. The experimental parameters were set as follows: a voltage of 400 V, scanning speed of 240 nm/min, and slit of 5 nm.

### 2.10. Meringue Batter Preparation

The freeze-dried powder was dissolved in deionized water to form a 10% solution. The solution was magnetically stirred for 2 h at room temperature and then refrigerated overnight at 4 °C until it reached equilibrium. The meringue was made as previously described with minor modifications [28]. Using a mixer (E-1051, Shunran, Guangzhou, China), 40 mL of EWP was mixed at a constant speed of 6 for 1 min, and when the foam was basically firm, 15 g of icing sugar was mixed in turn. After all the sugar had been added, the samples were frozen for 2 min under the same conditions.

### 2.11. Meringue Batter Properties

#### 2.11.1. Foam Density

A fixed volume of foam was weighed using a cylindrical container (40.192 mL), being careful of bubble trapping during transfer. A metal straightedge was used to scrape away excess foam from the top to obtain an even flat surface. The mass of the fluid foam was recorded at room temperature, and the process was repeated four times. The density of the foam was determined according to the following equation:

(6) $$Batter\;density=\frac{mass\;of\;fluid\;foam}{Cylindrical\;container\;volume}$$

#### 2.11.2. Rheology of the Meringue

The rheological behavior of the meringue was measured at 25 °C using a MARS40 rheometer (Thermo Scientific, USA). It was equipped with a P35/Ti probe (35 mm diameter) with a gap set to 1 mm [29].

The apparent viscosity of the meringue was measured as a function of the shear rate (1–100 s^−1^) and fitted via Cross Equation (7) to further analyze its rheological behavior.

(7) $$\eta =\eta_{\infty }+\frac{\left(\eta_{0}-\eta_{\infty }\right)}{1+{\left(\alpha_{c}\gamma \right)}^{m}}$$

where η is the apparent viscosity; η_0_ is the zero-shear-rate viscosity; η_∞_ is the infinite-shear-rate viscosity; γ is the shear rate; α_c_ is the trans-temporal constant (and sometimes consistency), which has a temporal dimension; and m is the factorless index. Owing to the low value of η_∞_ for food polymer dispersions at actual concentrations, η_∞_ was ignored in the fitting process.

Scanning measurements were in the range of 0.1–10 Hz at 0.1% strain [30]. The storage modulus (G′) and loss modulus (G”) of the meringue were measured. The power-law equation was as follows:

(8) $$G^{\prime }=K_{2}\cdot \omega n_{2}$$

(9) $$G^{\prime \prime }=K_{3}\cdot \omega n_{3}$$

denotes the relationship between the modulus and angular frequency, where ω is denoted as angular frequency (rad/s), K_2_ and K_3_ are denoted as dimensionless constants, and n_2_ and n_3_ are dimensionless frequency indices.

The parameters of the three-stage thixotropy test (3ITT) were set at a fixed shear rate of 0.1 s^−1^ for 270 s, a stabilized shear rate of 10 s^−1^ for 90 s, and a fixed shear rate of 0.1 s^−1^ for 270 s [31].

### 2.12. 3D-Printed Meringue

Foam was printed via a syringe extruder printer (FOODBOT-S2, Hangzhou, China) from Hangzhou Shiyin Technology Co., Ltd. (Hangzhou, China). The material was extruded to bring the foam extrusion to a steady state before formal printing. Printing parameters were set as follows: a nozzle diameter of 1.2 mm, print rate of 25 mm/s, print temperature of 25 °C, and fill density of 100%. Suitable 3D model parameters were established via Cura software (version 15.02.1).

### 2.13. Statistical Analysis

All the measurements were performed three times, and the results are expressed as the means ± standard deviations. A one-way analysis of variance (ANOVA) was performed via the statistical software SPSS 26.0, and Origin 2021 was used to obtain data and plot graphs.

## 3. Results

### 3.1. Foaming Properties

**Figure 1:**
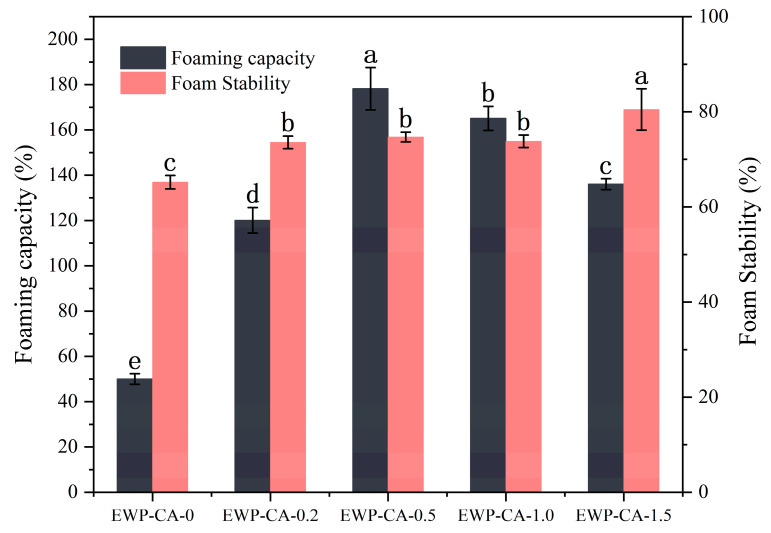
Foaming performance of EWP at different CA concentrations. Different letters indicate significant differences between data (*p* < 0.05).

Figure 1 shows the foaming properties of EWP particles at different citric acid concentrations. Compared with those of the control group, the foaming properties of the EWP particles were improved in all CA treatment groups. In particular, sample EWP-CA-0.5 had the highest foaming capacity, which was 3.56 times greater than that of the control. This occurred because as the concentration of citric acid increased, the acidic environment increased, and the molecular repulsion increased, leading to a greater degree of unfolding of the structure of the EWP. As a result, the flexibility and looseness of the protein structure were enhanced. Proteins with flexible structure accelerated their diffusion and adsorption at the air–water interface [32]. Under extreme conditions, the ionic strength increased, the protein structure changed, and some of the interactions of the protein side chains were lost. This led to the easy diffusion of proteins in the solution, thus increasing the potential for interfacial adsorption [33]. In addition, the lower viscosity of EWP-CA-0.5 facilitates the incorporation of air bubbles and the diffusion of molecules, thus increasing the efficiency of protein adsorption [8]. Notably, samples EWP-CA-1.0 and EWP-CA-1.5 had reduced foaming capacities. Because a large amount of CA unfolds the spatial structure of the EWP on a large scale, refolding upon pH adjustment reduces the degree of expandability of the protein, leading to reduced foaming properties [8]. In addition, its high viscosity prevented the fluid from passing through the liquid film network, whose behavior dominates, and the rate of protein adsorption at the air–water interface was reduced again, leading to a decrease in foaming. In terms of foam stability, the EWP from all CA treatment groups presented better foam stability. Among them, EWP-CA-1.5 had the best foam stability, which was attributed to the addition of CA, which allowed the protein to form a highly elastic film at the air–water interface with solid-like behavior. This prevented the drainage, disproportionation, and coalescence of the foam [28]. In addition, under extremely acidic conditions, protein aggregation occurred and liquid viscosity increased, which might affect the fluidity of the protein and its ability to diffuse across the interface, but increased the viscoelasticity of the interface, thus providing better foam stability. A similar trend was found by Wang et al., who characterized the proteins after using hydrochloric acid to create an extreme environment to induce the proteins into a molten state and found that the protein foaming properties were enhanced and the structural properties were improved [9]. From the characterization results, citric acid induced egg white proteins to have a higher foaming effect; in addition, citric acid-treated egg white proteins had lower interfacial tension to retard foam thinning. Therefore, the use of citric acid is safer and more efficient compared to hydrochloric acid.

### 3.2. Microscope Images

**Figure 2:**
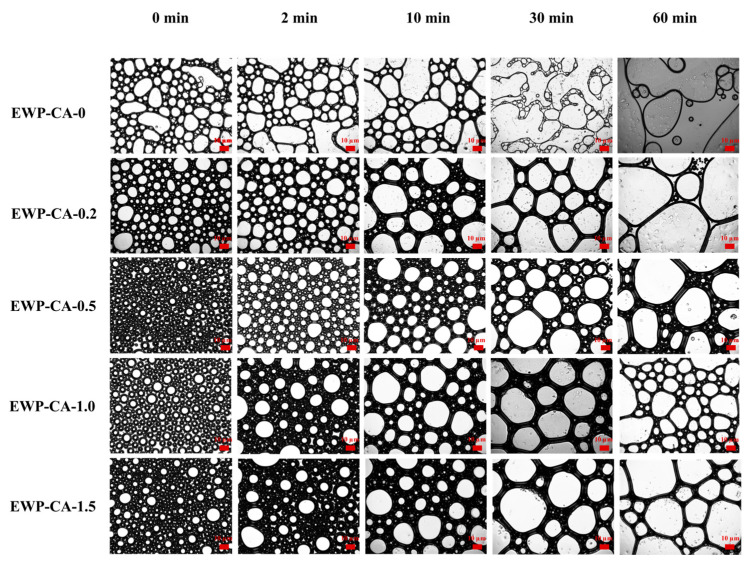
Images of foams with different CA concentrations treated with EWP at 4 × objective, 25 °C.

A microscopy image analysis was used to visually observe the effects of citric acid treatment on the properties of the EWP foams. Typically, foam properties are influenced by the shape, size, and density of the foam [34]. As shown in Figure 2, the diameter of the bubbles gradually increased with time, and the bubble morphology changed from spherical to polygonal bubbles. Because aqueous-phase bubbles are not thermodynamically stable, the liquid is expelled by gravity, resulting in thinning of the liquid film, which causes adjacent bubbles to merge [35]. At 2min, with the increase in CA concentration, the bubble distribution under the microscope gradually became uniform, and the bubble size gradually decreased (EWP-CA-0.2, EWP-CA-0.5), and the sample EWP-CA-0.5 had the best effect. Smaller bubbles can cluster together, which facilitates the formation of a deformation-resistant structure, resulting in a foam with greater drainage stability [36]. Compared with the control group, the EWP-CA sample group presented a significant reduction in foam rupture at 30 min but an increase in foam size. One of the samples, EWP-CA-0.5, had a smaller bubble size, the thickest liquid film wall, and a higher bubble density than the other groups did, and most of the bubbles remained spherical after 30 min. Microscopy results revealed that the addition of CA had a modifying effect on the proteins and enhanced the foaming properties and that the best foaming properties of the proteins were achieved at a citric acid concentration of 5 mg/mL.

### 3.3. Liquid Viscosity

**Figure 3:**
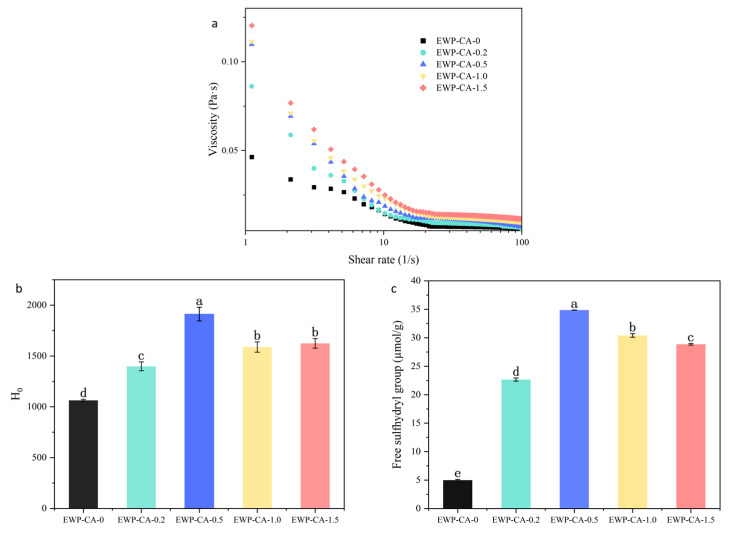
Liquid properties of EWP after reaction with different concentrations of CA are (**a**) liquid viscosity, (**b**) surface hydrophobicity, and (**c**) free sulfhydryl group content. Different letters indicate significant differences between data (*p* < 0.05).

The apparent viscosity of the liquid phase affects the fluidity of the continuous phase around the foam, thereby affecting the drainage rate of the foam [37]. As shown in Figure 3a, the viscosity of all samples decreased as the shear rate increased, which was typical of the shear-thinning behavior of non-Newtonian fluids. The trend of viscosity with the shear rate could reflect the steady-state shear properties of the liquid [22]. The EWP solution exhibited the lowest apparent viscosity at the same shear rate. Therefore, it failed to retain air during the bubble formation process, and the difficulty in forming a liquid surface film made it difficult to gather bubbles, leading to increased liquid discharge [38]. The apparent viscosity gradually increased with increasing CA concentration (EWP-CA-0.2, EWP-CA-0.5). Higher-viscosity samples increased foam stability because the increase in viscosity slowed the flow of liquid near the lamellae, retarding foam drainage and the thinning rate of the liquid film. As the concentration of citric acid continued to increase (EWP-CA-1.0, EWP-CA-1.5), the viscosity continued to increase due to the aggregation of proteins. Protein solutions with excessively high viscosity had a reduced foaming capacity because the higher initial viscosity prevented the fluid from passing through the liquid film network and affected the diffusion of the molecules, which reduced the protein adsorption rate and thereby affected the foaming properties of the protein [39]. In addition, a viscosity that is too high could lead to a low foaming capacity during the beating process, which is not conducive to the incorporation of air or the rapid spreading and unfolding of proteins near the interface [2].

### 3.4. Surface Hydrophobicity

Surface hydrophobicity can reflect the degree of unfolding of CA-treated egg white proteins, as well as the degree of exposure of hydrophobic groups [40]. As shown in Figure 3b, the surface of EWP without CA treatment was the least hydrophobic, which was related to the inability of ANS to enter the hydrophobic region of the protein [9]. The surface hydrophobicity of EWP increased significantly with increasing CA concentration. This result suggested that the natural structure of egg white protein was slightly altered during this process and that the number of hydrophobic sites available for ANS binding increased [41]. The formation of a protein adsorption layer played an important role in the construction and stabilization of the foam. Proteins need to diffuse into the air–water interface, thereby changing to the adsorbed state. Faster protein diffusion and adsorption enhance the foaming capacity, while the adsorption layer of proteins is influenced mainly by surface hydrophobicity [42]. Higher hydrophobicity promoted protein absorption at the interface [8]. Therefore, EWP treated with certain CA concentrations (EWP-CA-0.2, EWP-CA-0.5) exposed more hydrophobic groups, which enhanced the adsorption of proteins to the interface and inhibited the disproportionation of the foam, which in turn yielded a more robust foam structure. The surface hydrophobicity decreased as the CA concentration (EWP-CA-1.0, EWP-CA-1.5) continued to increase. This might be because, under more extreme conditions, some interactions within the MG were disrupted, and proteins aggregated, leading to hiding of hydrophobic groups and a consequent increase in the adsorption barrier at the air–water interface. The high hydrophobicity of the samples is one of the reasons for the high foaming capacity, and this hydrophobicity is consistent with the trend in foaming properties.

### 3.5. Free Sulfhydryl Content

Disulfide bonds play a key role in the stabilization of egg white protein conformation [43]. The activity of the air–water interface and ability to form protein adsorption layers also depend to some extent on the conformation of the interfacial proteins, so it is important to understand changes in disulfide bonds [44]. The effect of citric acid treatment on free sulfhydryl content of EWP is shown in Figure 3c. The free sulfhydryl content of egg whites with different levels of acidification significantly increased, and the free sulfhydryl content of each group of samples was 4.4 times (EWP-FA-0.2), 6.8 times (EWP-FA-0.5), 6 times (EWP-FA-1.0), and 5.6 times (EWP-FA-1.5) greater than that of EWP, respectively. Among egg white proteins, ovalbumin is the only protein with a free sulfhydryl group, which is connected by a single disulfide bond between Cys74 and Cys121 [45]. Extremely acidic conditions might disrupt disulfide bonds within the protein molecule, leading to an increase in free sulfhydryl content. Liu et al. reported that the disruption of disulfide bonds led to unfolding of the protein structure, thereby exposing free sulfhydryl groups to solvents [46]. The presence of disulfide bonds reduces the flexibility of proteins and thus improves the properties of foam films [47]. Sample EWP-FA-1.0, with EWP-CA-1.5, presented a decreasing trend in free sulfhydryl content, which might be due to the extremely acidic thermal oxidation of the exposed free sulfhydryl groups, resulting in a decrease in their amount.

### 3.6. Surface Tension

**Figure 4:**
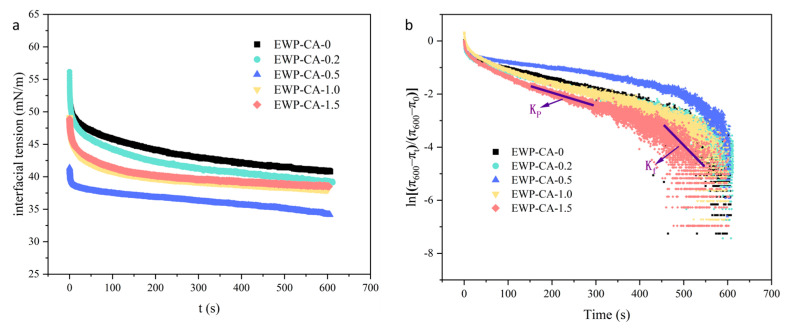
The air–water interface properties of EWP at different CA concentrations: (**a**) dynamic interfacial tension, (**b**) time dependence of ln[(π_600_ − π_t_)/(π_600_ − π_0_)].

Air–water interfacial tension reflects the rate of protein adsorption and swelling at the air–water interface [34]. As shown in Figure 4a, the interfacial tension decreases with the increase in time. The rapid decrease in interfacial tension in all the groups indicates that protein molecules adsorb and diffuse rapidly at the air–water interface. The significant reduction in surface tension facilitates a better migration of proteins to the air–water interface for adsorption, stretching, and orientation to form a layer upon the layer of film, which significantly improves the foaming capacity [48]. The decrease rate of EWP interfacial tension gradually increased with the increase in citric acid concentration, and the interfacial tension of EWP-CA-0.5 was the lowest. In the process of acidic environment enhancement, the EWP surface charge and molecular repulsion gradually increased, the protein structure opened, and molecular flexibility increased. Proteins with high surface hydrophobicity with a molten globule conformation can migrate and adsorb to the air–water interface more rapidly [49]. The addition of citric acid changed the structure of egg white protein, allowing it to adsorb more quickly at the air–water interface, leading to a rapid decrease in interfacial tension. As the CA concentration continued to increase (EWP-CA-1.0, EWP-CA-1.5), the strong acid environment denatured the structural unfolding of the molten globule, and the decreasing trend of the interfacial tension slowed. This might be due to the emergence of protein aggregates, which affects the mobility and accessibility of proteins [32].

After proteins diffuse to the air–water interface, they enter a permeation and rearrangement phase, which controls the rate of adsorption [50]. Figure 4b shows that there were two linear regions of the time-ln[(π_600_ − π_t_)/(π_600_ − π_0_)] curve. The permeability rate constant K_p_ and the rearrangement rate constant K_f_ were determined via linear fitting (Table 1). The K_p_ and K_f_ of the EWP-CA samples were greater than those of the control, indicating a better permeation and rearrangement of the CA-treated EWP samples. The permeation process is usually related to factors such as the molecular structure of EWP and CA and the interfacial affinity. In extreme environments, CA induced unfolding of the EWP structure, resulting in an overall flexible and loose conformation. This structure reduced the energy potential for EWP-CA adsorption at the interface, thereby enhancing interfacial adsorption and accelerating sample permeation at the air–water interface [51]. When the citric acid concentration was too high (EWP-CA-1.0, EWP-CA-1.5), the permeation rate decreased, which might have been due to the existence of hydrophobic interactions between CA and EWP, which blocked the hydrophobic structural region and was therefore unfavorable for the permeation of the samples at the air–water interface [52]. During the recombination phase, elevated K_f_ indicates that the protein could quickly adapt to the interfacial environment and undergo rapid conformational recombination at the interfacial layer [53]. The strong electrostatic repulsion (charged groups interacting with water) in polar environments led to reduced molecular entanglement in the protein structure, resulting in a stronger drive for interfacial remodeling, which promoted the rearrangement of molecules. However, when the CA concentration was too high (EWP-CA-1.0, EWP-CA-1.5), many proteins aggregated in the interfacial layer, limiting the interfacial movement of the proteins and preventing necessary structural transformations; consequently, the rate of rearrangement decreased. Therefore, an appropriate CA could accelerate permeation and rearrangement rates of EWP at the air–water interface and stabilize the interface.

### 3.7. Spectral Analysis

**Figure 5:**
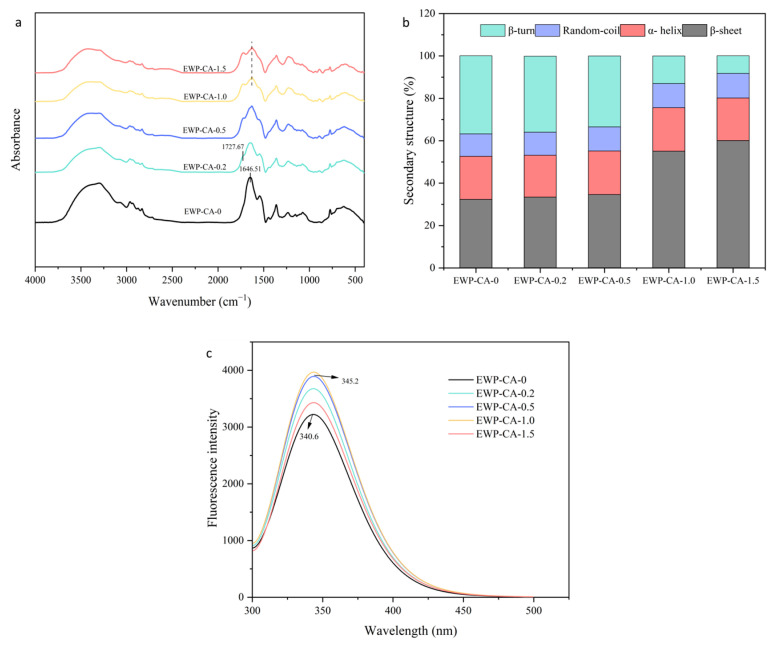
Structural properties of EWP at different CA concentrations: (**a**) FIRT spectra, (**b**) the calculation of the percentage of the secondary structure of egg white protein from the results of the FIRT spectra, (**c**) fluorescence spectra.

FTIR spectroscopy has been used to characterize the interactions between protein parts and to analyze changes in the secondary structure of proteins [54]. For different states of EWP-CA, a new characteristic peak appeared at 1727.67 cm^−1^, which was attributed to the carboxylic acid group and the ester carbonyl C = O stretching vibration. The peak at 1646.51 cm^−1^ was the amide I band of the sample (1600–1700 cm^−1^), and those of EWP-CA-0, EWP-CA-0.2, and EWP-CA-0.5 were not blueshifted or redshifted (Figure 5a). When the CA concentration continued to increase (EWP-CA-1.0, EWP-CA-1.5), the peak of the amide I band redshifted, and the intensity of the EWP peak and position of the peak of the amide I band were altered, which might be the result of protein side chain rearrangement and protein aggregation in the extremely acidic environment [55]. The amide I band is the most sensitive spectral region for the protein secondary structure, which mainly originates from the C = O stretching vibration of protein peptide bonds, and its fitting can yield information on the protein spatial structure. For samples EWP-CA-0, EWP-CA-0.2, and EWP-CA-0.5, the results fitted to the analysis of the secondary structure revealed that there was no significant change in the percentage of each group (Figure 5b). Therefore, EWP treated with certain CA concentrations still retained a secondary structure comparable to that of the natural state, allowing the protein to form MG in a more flexible state as a way to improve functional properties of EWP. Similar results were obtained by Wang et al., who reported that the secondary structure of soybean isolate proteins was not altered but that the tertiary structure was disrupted after they were induced to the MG state in a polar environment [56]. When the CA concentration (EWP-CA-1.0, EWP-CA-1.5) was increased, α-helix and irregular coil contents of samples were relatively stable, but β-folding content increased, whereas the β-turn content decreased with increasing citric acid concentration, which indicated that a certain degree of aggregation occurred in proteins [57], which led to increased gelation enhancement in the samples instead of increased interfacial properties.

Fluorescence spectroscopy is the usual method for probing tertiary structural changes in proteins. The intrinsic fluorescence of proteins is caused by aromatic amino acids such as tryptophan (Trp), tyrosine (Tyr), and phenylalanine (Phe). The fluorescence characteristics of the samples are shown in Figure 5c, where the maximum fluorescence intensity redshifted as the concentration of citric acid increased (340.6–345.2). This phenomenon suggested that the structure of the egg white protein was opened and the increased polarity of the microenvironment of the tryptophan residues led to an increased exposure of the residues, thereby altering the conformation of the tryptophan and leading to the disruption of the tertiary structure of the protein [58]. The fluorescence intensity tended to increase with increasing citric acid concentration (0–10 mg/mL). This suggested that the addition of citric acid affected the conformation around the tryptophan, leading to a decrease in the intramolecular quenching of tryptophan residues and an increase in fluorescence intensity [59]. In addition, acidic environments might affect the charge density of proteins. The repulsive forces between molecules in highly charged regions are strong enough to induce protein defolding. When proteins are highly defolded, exposing reactive groups such as hydrophobic groups or sulfhydryl groups, intermolecular sulfhydryl/disulfide bond exchange within protein membranes and protein adsorption at air–water interfaces are accelerated [60]. Notably, the maximum fluorescence intensity was reduced at a citric acid concentration of 15 mg/mL, which might be due to the fact that acid heat treatment might lead to microenvironmental changes in the oxidation of tryptophan residues in complex food systems [61]. In addition, in a high-intensity acidic environment, protein aggregation will also lead to fluorescence quenching.

### 3.8. Meringue Ink Properties

Meringue (sweetened egg white foam) is a key component of bakery products, including macarons, mousse cake, tiramisu, etc., and provides firm structural support for bakery products [62]. Owing to the simplicity of its composition, the rheological properties of meringue were used as a model system to study the behavior of the unfolding and folding of the EWP structure in an aeration system containing high levels of sugars as a result of citric acidification treatment. EWP-CA-0 was selected as the control group, and EWP-CA-0.2, EWP-CA-0.5, and EWP-CA-1.0 were selected as the experimental groups for the preparation of meringue batters to study their batter properties.

#### 3.8.1. Batter Density

**Figure 6:**
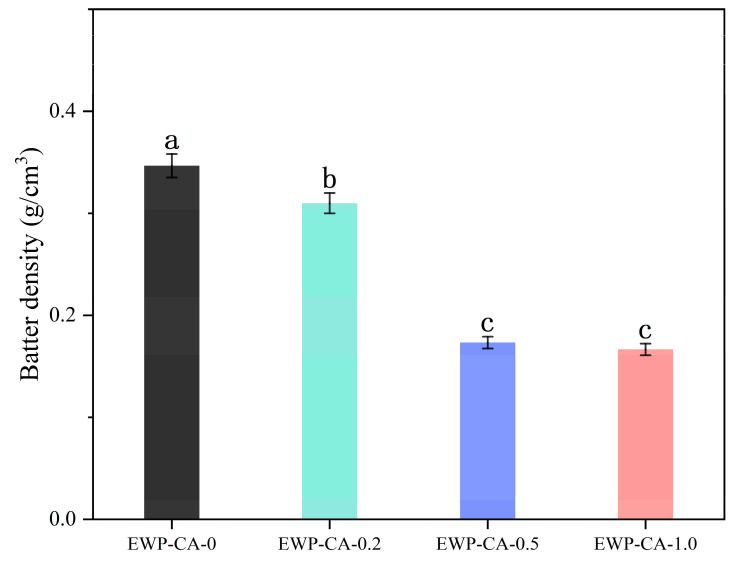
Density values of meringue under different CA concentration treatments. Different letters indicate significant differences between the data (*p* < 0.05).

Figure 6 shows the values of protein meringue density produced by the control sample and the experimental group of citric acid-treated EWP. In this study, the meringue density at zero citric acid addition was 0.35 g/cm^3^, and the EWP-CA-0.2 meringue density was 0.31 g/cm^3^. In EWP-CA-0.5 and EWP-CA-1.0, there was a significant and substantial reduction in the density values of samples, which were statistically significant at 0.17 g/cm^3^ and 0.16 g/cm^3^ (*p* < 0.05), respectively. Meringue comes from the protein foam formed by whipping egg whites. In the sample set, the density value of meringue decreased significantly as the concentration of citric acid increased, which was due to the expansion of the bubbles in the protein system, and the volume of the air phase increased, resulting in a decrease in the density value of meringue [63]. The low density value reflected greater foam overflow and better foam performance of the samples, indicating better performance in terms of the volume and quality of the meringue from the citric acid-treated egg whites. The results of the meringue density analysis revealed that citric acid treatment could change the foam structure and thus enhance protein function. EWP plays a very important role in the foaming system of the food industry. Various methods have been used to improve EWP foaming properties. Citric acid treatment of egg white proteins reduced foam density and increased the air phase, which could be used in the production of meringues to increase yields.

#### 3.8.2. Foam Rheology

**Figure 7:**
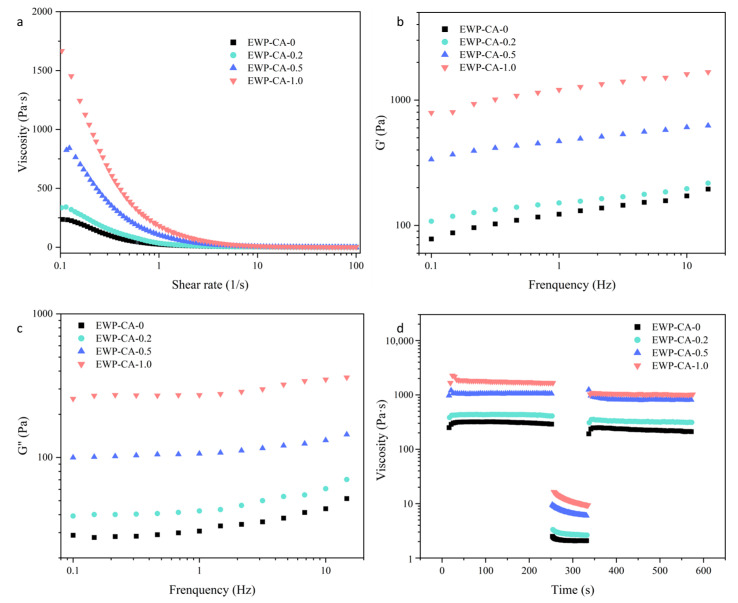
Rheological properties of meringue under different CA concentration treatments: (**a**) viscosity, (**b**,**c**) frequency scan, (**d**) shear recovery.

Shear-thinning behavior reduces the pressure required by the machine to extrude the ink, a material property required for 3D printing [64]. The apparent viscosity of all meringue samples decreased rapidly in the range of shear rates from 1 to 100 s^−1^, and their steady-state flow profiles matched those of structured fluids with pronounced shear-thinning behavior (Figure 7a). This suggested that the stretching of the foam structure under shear forces reduced network entanglement, leading to a reduction in local resistance and hence viscosity. To further analyze the effect of flow behavior on meringue 3D printing, the apparent viscosity data were fitted via the Cross model. As shown in Table 2, the Cross model showed a strong correlation (R2 ≥ 0.98) and therefore could describe the rheological behavior well. α_c_ is the cross-time constant (sometimes called consistency) with a time dimension of 1/α_c_, which represents a critical shear rate and is a standard for the onset of the shear rate in the shear-thinning region [65]. The fitting results revealed that the α_c_ constant of the sample group with the addition of citric acid progressively increased, indicating that the shear-thinning region appeared at lower shear rates in the sample group than in the control group without citric acid. m is a key parameter for dependence of viscosity on the shear rate in the shear-thinning region, and the larger the value of m is, the stronger the shear-thinning property of the material. The m-parameter of the citric acid-containing EWP-CA meringue increased with increasing citric acid concentration, indicating that the citric acid-added meringue had stronger shear-thinning properties than did the natural protein frosting, especially for sample EWP-CA-0.5. η_0_ is the zero-shear-rate viscosity, and the η_0_ values of meringues made from EWP-CA-1.0 and EWP-CA-0.5 were 4.7 and 3.7 times greater than those of the control without citric acid treatment. The high zero-shear viscosity of the sample meringue was partly related to the molten globule state of the proteins [8]. The MG state had a secondary structure and intrinsic viscosity similar to that of the natural state and possessed an extended tertiary structure determined by peptide side chains, with a more convoluted morphology in which the radius of the morphology would be extended by approximately 10–30% compared with its natural state. The variation in EWP morphology gave the meringue a high viscosity. In addition, under high-acid conditions, the aggregation of proteins also led to an increase in viscosity. However, as the shear rate increases, the entanglement opens and stretches in the direction of flow, leading to a decrease in physical interactions [28]. Therefore, the group of EWP-CA-0.5 meringue samples with higher m and α_c_ values had better and earlier shear-thinning behavior. Meringues with strong shear-thinning properties and high viscosity facilitate the extrusion and 3D printing of confectionery products, as they can be easily extruded from the nozzle at low shear and quickly cured again after exiting the nozzle [66]. On the other hand, there was competition between the sugar powder and protein molecules for binding with water. Therefore, in the presence of powdered sugar, protein-water interactions do not dominate thermodynamically, which also leads to increased hydrophobic interactions between proteins [67]. In view of the results of the hydrophobicity study described above, the citric acid-treated EWP particles presented better hydrophobic properties. It could be expected that EWP-CA-0.5 produces more hydrophobic interactions between proteins at high sugar concentrations. This would greatly improve its foam overflow and increase the yield of the meringue.

To analyze the oscillatory response of the meringue, it was frequency scanned in the frequency range of 0.01–10 Hz. The results of frequency scans of different meringues are shown in Figure 7b,c. The storage modulus (G′) represents the elasticity of the solid, the loss modulus (G′′) represents the viscosity of the liquid, and the frequency-dependent relationship between G′ and G′′ can determine the structural type of the meringue. For all the meringues, G′ was always much larger than G′′, indicating that all samples had a gel network structure with elastic gel properties. To quantify the degree of frequency dependence of the storage modulus (G′) on the loss modulus (G′′), a power-law model was fitted to experimental results of the meringue oscillation scans, and parametric results are shown in Table 2. The coefficients K and *n* denote the size of the intercept at a frequency of 1 Hz and slope of the modulus as a function of frequency (ω). The power-law constant K is commonly used to describe the range of elastic and viscous properties of fluids [68]. The power-law constants K_2_ and K_3_ increase significantly with increasing CA, indicating that CA improved the elastic and viscous behavior of meringue. *n* denotes the frequency dependence of the modulus; when the value of *n* is close to zero, the material is characterized by a solid-like state, and when *n* is 1, the system behaves as a viscous material [69]. The decreased n_1_ and n_2_ values after the addition of CA indicate an improvement in the stability of the meringue structure. However, when the CA concentration (EWP-CA-1.0) was elevated to a certain concentration, the *n* value increased, which could be attributed to the destruction of the internal structure caused by excess CA. Excessive viscosity also reduced the structural stability of the meringue [68].

As the meringue passes through the 3D printer nozzle, the extruded icing needs to have good mechanical properties to complete the shape stack. Therefore, the shear recovery values of the samples can be used as indicators of the performance of the mechanical properties of meringue, which underwent the whole printing process. The shear recovery of the meringue is shown in Figure 7d at a shear rate of 0.1 s^−1^, simulating the flow behavior of the sample before printing. The samples had a good viscosity steady state, indicating good structural support of meringue. At shear rates up to 10 s^−1^, the process of sample extrusion from the nozzle while printing was simulated. The sudden decrease in viscosity at this stage could be attributed to the destruction of the high-density structure of the sample due to squeezing of the sample by the nozzle. The subsequent recovery of the sample viscosity value at a shear rate of 0.1 s^−1^ also demonstrated that the excellent mechanical properties of CA-treated meringue could ensure the coherence of the multilayer stacks. Similar results were obtained by Hou et al. [70]. Notably, the viscosity of the EWP-CA-1.0 meringue samples tended to decrease after experiencing a high shear rate (10 s^−1^) and did not return to values below the low shear rate (0.1 s^−1^), which might be attributed to the disruptive effect of CA on the structure of samples themselves in a certain concentration range; therefore, the mechanical properties decreased. The results showed that the EWP-CA-0.5 samples had better mechanical properties and could recover their network structure well after experiencing high shear speeds, which provided a good basis for subsequent 3D printing and helped the samples maintain their initial properties after extrusion.

### 3.9. Meringue 3D Printing

**Figure 8:**
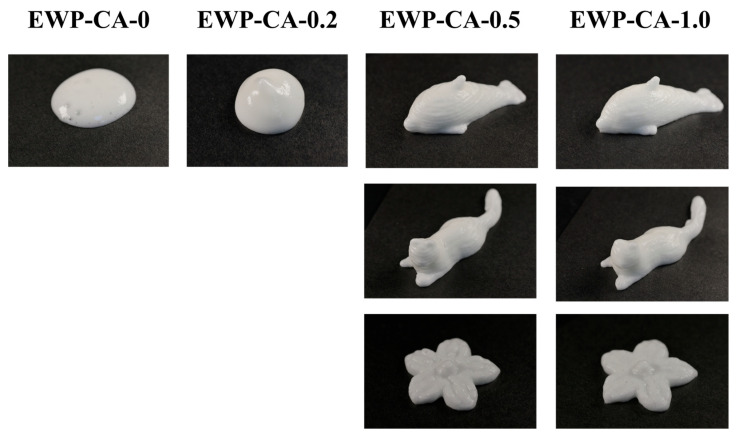
Three-dimensional printing performance of meringue under different CA concentration treatments.

The problem with printing food materials with foamy properties is the lack of material rigidity, which leads to the collapse of the sample in a short period of time, especially if density changes during the printing process [71]. Therefore, it is important that the food material is homogeneous in the 3D printing process and has good mechanical properties with continuous fluid properties [4]. Citric acid-modified EWP was printed into different geometries to verify the feasibility of meringue in 3D food material printing applications, and the results are shown in Figure 8 below. Samples EWP-CA-0 and EWP-CA-0.2 did not show good support properties. Compared with the flatness of the EWP-CA-0 sample, the EWP-CA-0.2 sample had a visible height and a smoother meringue surface. This result suggested that there was some degree of structural improvement in the EWP after treatment with low concentrations of CA. The ideal state of 3D printing is high fidelity in the shape of the material, the consistent and smooth extrusion of the sample without extrusion distortion during the printing process, and precise attachment to the previously deposited layer [72]. Both the EWP-CA-0.5 and the EWP-CA-1.0 print firm geometries, but as can be seen from the picture in Figure 8, the EWP-CA-0.5 had a much better form, with clearer print layers and a smoother surface. The storage modulus G′ reflects mechanical properties of material and is a key indicator of the material’s dimensional consistency with previously deposited layers. EWP-CA-0.5’s higher G′ indicates high resolution and high print precision [73]. On the other hand, proper shear-thinning behavior could help the extrusion of the fluid material from the nozzle, benefiting the deposition behavior during the printing process [74]. Sample EWP-CA-1.0 suffers from blurred print layers and rough surfaces, and it is also evident from the image that it had poor structural compactness and a puffy shape for the same amount of ink output. As a result, the EWP-CA-0.5 meringue had high mechanical properties with strong shear-thinning behavior, and as expected, it showed better 3D printing performance.

## 4. Conclusions

In this study, an ink consisting of citric acid-modified EWP was developed and applied to produce 3D-printed meringue. CA treatment of EWP shifted to the MG state and significantly affected its functional properties. The protein viscosity increased appropriately, slowing the liquid flow and thus delaying foam drainage. An increase in free sulfhydryl content increased pliability of protein and improved properties of the foam film. In addition, proteins with high surface hydrophobicity can more rapidly adsorb to the air–water interface, leading to a rapid decrease in interfacial tension. The secondary structure of natural proteins remains essentially unchanged after unfolding and folding. However, the tertiary structure is altered, mainly involving the exposure of amino acid residues with the expansion of hydrophobic regions. The change in protein structure resulted in a dramatic improvement in foaming properties. The improvements in the fluidic and foam properties of the meringue were strongly correlated with the alterations in structural properties of EWP caused by CA. The shear-thinning behavior and storage modulus G′ exhibited by meringue during 3D printing were key factors for three-dimensional geometries to have high resolution and resistance to structural collapse. These findings suggested that CA-treated EWP has improved foaming properties and has the ability to act as a meringue weaving agent, and its stability in food 3D printing indicates that these edible inks and printed foods can manufacture more personalized foods in the future, promoting the development of 3D-printed foods. In this study, we succeeded in developing novel meringue formulations that give high yields, and we demonstrated their applicability in 3D printing. The application direction of this study is mainly in meringue, but it has not been extended to other baking products, and there is no in-depth discussion on food flavor and sensory aspects. In the future, further research on different foam products and product flavors can be conducted to develop more novel edible foams.

## Figures and Tables

**Table 1:** Characteristic kinetic parameters of EWP adsorption at air–water interface under different CA concentration treatments, permeation rate (K_p_) and rearrangement rate (K_f_).

Sample	K_p_ × 10^3^ (s^−1^)	K_f_ × 10^2^ (s^−1^)
EWP-CA-0	−4.21 ± 0.27 ^b^	−1.39 ± 0.84 ^c^
EWP-CA-0.2	−4.53 ± 0.24 ^b^	−1.94 ± 0.75 ^b^
EWP-CA-0.5	−6.32 ± 0.20 ^a^	−5.88 ± 0.82 ^a^
EWP-CA-1.0	−4.89 ± 0.33 ^b^	−2.29 ± 1.14 ^b^
EWP-CA-1.5	−4.45 ± 0.25 ^b^	−0.96 ± 1.28 ^c^

Different letters indicate significant differences between data (*p* < 0.05).

**Table 2:** Results of model fitting for the rheological behavior of the meringue under different CA concentration treatments.

Model	Model Parameters	EWP-FA-0	EWP-FA-0.2	EWP-FA-0.5	EWP-FA-1.0
Cross $\eta =\eta_{\infty }+\frac{\eta_{0}-\eta_{\infty }}{1+{\left(\alpha_{c}\gamma \right)}^{m}}$	η_0_ (Pa⋅s)	2.46 × 10^2^	3.48 × 10^2^	9.15 × 10^2^	11.66 × 10^2^
	α_c_ (s)	3.42	3.62	3.77	3.75
	m	1.95	1.98	2.15	2.07
	R^2^	0.98	0.98	0.98	0.98
Power-law $G^{\prime }=k_{2}\omega^{n_{2}}$	k_2_ (Pa)	1.21 × 10^2^	1.49 × 10^2^	4.65 × 10^2^	11.73 × 10^2^
	*n*	0.16	0.12	0.11	0.14
	R^2^	0.98	0.98	0.99	0.98
Power-law $G^{\prime \prime }=k_{3}\omega^{n_{3}}$	k_3_ (Pa)	32.8	45.5	110.8	288.8
	*n*	0.13	0.12	0.07	0.07
	R^2^	0.98	0.98	0.98	0.98

## Data Availability

The original contributions presented in this study are included in the article. Further inquiries can be directed to the corresponding author.
